# Monitoring antimalarial safety and tolerability in clinical trials: A case study from Uganda

**DOI:** 10.1186/1475-2875-7-107

**Published:** 2008-06-11

**Authors:** Sarah G Staedke, Prasanna Jagannathan, Adoke Yeka, Hasifa Bukirwa, Kristin Banek, Catherine Maiteki-Sebuguzi, Tamara D Clark, Bridget Nzarubara, Denise Njama-Meya, Arthur Mpimbaza, Philip J Rosenthal, Moses R Kamya, Fred Wabwire-Mangen, Grant Dorsey, Ambrose O Talisuna

**Affiliations:** 1 London School of Hygiene & Tropical Medicine, London, UK; 2 c/o MU-UCSF Research Collaboration, Mulago Hospital Complex, P.O. Box 7475, Kampala, Uganda; 3 Department of Medicine, San Francisco General Hospital, University of California, San Francisco, USA; 4 Uganda Malaria Surveillance Project, Kampala, Uganda; 5 Makerere University Medical School, Kampala, Uganda; 6 Makerere University, School of Public Health, Kampala, Uganda; 7 Ministry of Health, Kampala, Uganda

## Abstract

**Background:**

New antimalarial regimens, including artemisinin-based combination therapies (ACTs), have been adopted widely as first-line treatment for uncomplicated malaria. Although these drugs appear to be safe and well-tolerated, experience with their use in Africa is limited and continued assessment of safety is a priority. However, no standardized guidelines for evaluating drug safety and tolerability in malaria studies exist. A system for monitoring adverse events in antimalarial trials conducted in Uganda was developed. Here the reporting system is described, and difficulties faced in analysing and interpreting the safety results are illustrated, using data from the trials.

**Case description:**

Between 2002 and 2007, eleven randomized, controlled clinical trials were conducted to compare the efficacy, safety, and tolerability of different antimalarial regimens for treatment of uncomplicated malaria in Uganda. The approach to adverse event monitoring was similar in all studies. A total of 5,614 treatments were evaluated in 4,876 patients. Differences in baseline characteristics and patterns of adverse event reporting were noted between the sites, which limited the ability to pool and analyse data. Clinical failure following antimalarial treatment confounded associations between treatment and adverse events that were also common symptoms of malaria, particularly in areas of lower transmission intensity.

**Discussion and evaluation:**

Despite prospectively evaluating for adverse events, limitations in the monitoring system were identified. New standardized guidelines for monitoring safety and tolerability in antimalarial trials are needed, which should address how to detect events of greatest importance, including serious events, those with a causal relationship to the treatment, those which impact on adherence, and events not previously reported.

**Conclusion:**

Although the World Health Organization has supported the development of pharmacovigilance systems in African countries deploying ACTs, additional guidance on adverse events monitoring in antimalarial clinical trials is needed, similar to the standardized recommendations available for assessment of drug efficacy.

## Background

Since 2004, antimalarial treatment in sub-Saharan Africa has changed dramatically. As a result of widespread resistance to chloroquine and sulphadoxine-pyrimethamine, most sub-Saharan African countries have recently revised their antimalarial drug policies, selecting new artemisinin-based combination therapies (ACTs) as first-line treatment for uncomplicated malaria [1]. Although these drugs appear to be safe and well-tolerated, experience with their use in Africa is limited and continued assessment of ACT safety is a priority [2]. Artemether-lumefantrine and artesunate-amodiaquine were initially chosen as first-line regimens in most African countries [3]. However, several additional regimens, including dihydroartemisinin-piperaquine, have recently been introduced and are being considered for widespread use [4-6]. With increasing availability of highly effective ACTs, selection of the optimal regimen will be driven by factors other than efficacy, including safety and tolerability, ease of administration, and cost. Comparative evaluations of the safety and tolerability of new regimens will become increasingly important for informing treatment decisions and policy.

Guidelines for assessing the efficacy of antimalarial treatments have been provided by the World Health Organization (WHO) [7-9]. However, although the importance of pharmacovigilance for antimalarial treatment has been increasingly recognized, there are no standardized recommendations for evaluating drug safety and tolerability in antimalarial trials [2,10,11]. Guidelines for laboratory testing are also lacking. In addition, few antimalarial studies publish detailed methods on the collection of safety data [5,12-15]. The lack of a standardized approach to adverse event monitoring limits the ability to compare data collected in different studies.

To evaluate the safety and tolerability of newer combination antimalarial therapies, a system for monitoring adverse events in clinical trials conducted in Uganda since 2002 was developed. This system has been used in studies evaluating treatment of single episodes of uncomplicated malaria, and in a longitudinal cohort study evaluating repeated antimalarial treatments. Here the reporting system is described, and difficulties faced in analysing and interpreting the safety data are discussed. The challenges of adverse event monitoring and key areas that need to be addressed in developing guidelines for assessing the safety and tolerability of antimalarial drugs are highlighted.

## Case description

### Study design

From 2002 to 2007, eleven randomized, controlled clinical trials were conducted to compare the efficacy, safety, and tolerability of different combination antimalarial regimens for treatment of uncomplicated malaria in Uganda (Table 1). Ten of the trials evaluated treatment of single episodes of malaria using a similar study design and a common protocol [5,15-18]. One trial was a longitudinal study of a cohort of children comparing three different combination regimens for treatment of repeated episodes of uncomplicated malaria [19,20,27].

**Table 1:** Summary of antimalarial clinical trials conducted in Uganda 2002 – 2007

**Trial**	**Kampala 1**	**UMSP 1**	**UMSP 2**	**UMSP 3**	**UMSP 4**	**Kampala 2**
Study site(s)	Kampala	Kyenjojo, Mubende, Kanungu	Jinja, Arua, Tororo, Apac	Tororo	Apac	Kampala

Years conducted	August 2002 – July 2003	December 2002 – June 2003	November 2002 – May 2004	December 2004 – July 2005	May – July 2006	November 2004 – May 2007

Regimens evaluated*	CQ+SP	CQ+SP	CQ+SP	AS+AQ	AL	AQ+SP
	AQ+SP	AQ+SP	AQ+SP	AL	DP	AS+AQ
	AS+AQ		AS+AQ			AL

Duration of follow-up	28 days	28 days	28 days	42 days	42 days	42 days
Age range of participants	0.5 – 10 years	≥ 6 months	≥ 6 months	1 – 10 years	0.5 – 10 years	1 – 10 years
Total study treatments	400	1105	2160	408	421	1120 †
Efficacy outcome assigned	384 (96%)	1057 (96%)	2081 (96%)	403 (99%)	417 (99%)	1108 (99%)

Total SAEs	16	41	20	2	6	18
Convulsions	4	15	4	1	3	9
Altered mental status	1	0	2	0	0	0
Anemia	5	6	3	0	0	0
Vomiting	2	13	1	0	0	1
Weakness	0	3	1	0	0	2
Respiratory illness	1	1	4	1	0	2
Other	3	3	5	0	3	4

* CQ+SP: chloroquine + sulphadoxine-pyrimethamine; AQ+SP: amodiaquine + sulphadoxine-pyrimethamine; AS+AQ: artesunate + amodiaquine; AL: artemether-lumefantrine; DP: dihydroartemisinin-piperaquine

† Of 602 children enrolled into the Kampala 2 cohort study, 382 were diagnosed with at least one episode of uncomplicated malaria and were treated with a total of 1120 study treatments.

Ethical approval for the studies was obtained from the Uganda National Council for Science and Technology (all studies); the University of California, San Francisco Committee on Human Research (all studies); the Makerere University Research and Ethics Committee (Kampala studies, and UMSP 4); and the University of California, Berkeley (UMSP 1, 2, and 3).

### Study sites

**Figure 1:**
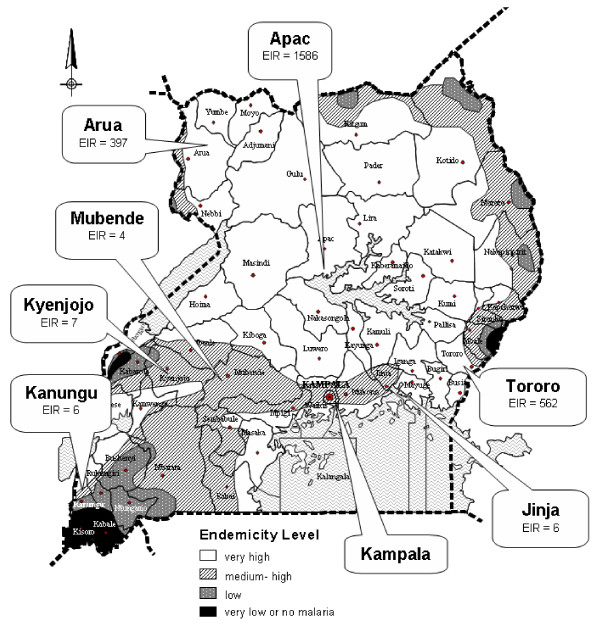
Study sites in Uganda and malaria endemicity.

Kampala is an urban center where malaria is meso-endemic (25% palpable spleen rate, 25% parasitaemia rate, Uganda Ministry of Health, unpublished data, 1994). The UMSP study sites, selected for geographic diversity, included: Kanungu (rural, low transmission), Kyenjojo (rural, medium transmission), Mubende (rural, medium transmission), Arua (rural, high transmission), Apac (rural, very high transmission), Tororo (rural, very high transmission), and Jinja (peri-urban, medium transmission) (Figure 1). The level of malaria transmission intensity at the UMSP sites has been further characterized by estimates of entomological inoculation rates (Figure 1) [21].

### Baseline clinical and laboratory evaluations

The study procedures for all eleven clinical trials have been previously reported [5,15-18,20]. On the day of malaria diagnosis (day 0), a standard clinical evaluation was performed, including history and physical examination. Guidelines for conducting the physical examination were provided (Additional File 1). In the initial studies (Kampala 1 and UMSP 1 and 2), a brief neurologic assessment, consisting of tests for coordination (heel-toe ataxia), fine finger dexterity (ability to pick up a small object), hearing, nystagmus, and balance (Romberg test), was conducted in appropriate aged children. In the later studies (UMSP 3 and 4, and Kampala 2), only fine finger dexterity and hearing were routinely evaluated.

Baseline laboratory evaluations in the Kampala studies included thick and thin blood smears, complete blood count, alanine aminotransferase (ALT), and creatinine (Kampala 1 only). In the UMSP studies, blood was obtained by finger-prick for thick and thin blood smears, and haemoglobin measurement.

### Antimalarial treatment allocation and blinding

Participants were randomly assigned study treatment in all trials, although procedures varied slightly between studies. Details on randomization, treatment allocation and administration, and blinding are included in the initial study reports [5,15-18,20]. For all studies, the only study personnel aware of the treatment assignments were the nurses responsible for allocating and administering treatment. Study participants were not informed of their assigned treatment regimen.

### Follow-up clinical and laboratory evaluations

The standardized history and physical examination was repeated at all scheduled and unscheduled follow-up visits. Follow-up laboratory assessments in the Kampala studies included thick blood smears, complete blood count and ALT (on days 7 and 28 in Kampala 1, and day 14 in Kampala 2), and creatinine (on days 7 and 28 in Kampala 1). In the UMSP studies, repeat assessments included thick blood smears, and haemoglobin on the final day of follow-up or the day of treatment failure.

### Adverse event monitoring

The approach to adverse event monitoring was similar in all studies. An adverse event was defined as any untoward medical occurrence, irrespective of its suspected relationship to the study medications as per International Conference of Harmonization (ICH) guidelines for Good Clinical Practice (GCP) [22]. A serious adverse event was defined as any adverse experience that was life threatening, or resulted in death, hospitalization, persistent or significant disability or incapacity, or required a specific medical or surgical intervention to prevent serious outcome.

At each participant encounter a standardized system was used to assess symptoms, physical exam findings, and laboratory test results based on grading of severity (Additional File 2). The severity grading scales were adapted from guidelines provided by the World Health Organization (Toxicity Grading Scale for Determining the Severity of Adverse Events) and the National Institutes of Health, Division of Microbiology and Infectious Diseases (Pediatric Toxicity Tables, May 2001). Active assessment for specific symptoms was conducted in all participants, including weakness, anorexia, vomiting, diarrhoea, cough, pruritus, and coryza. Headache, abdominal pain, and nausea were also assessed in children over three years of age. Any additional symptoms reported by the participant or findings noted in physical exam were assessed and graded accordingly.

Adverse events were identified by evaluating for any new or worsening symptoms, physical exam findings, or laboratory abnormalities. An adverse event was identified based on an increase in the severity score as compared to the previous evaluation. Participants with abnormalities present at baseline (on day 0) would not be classified as experiencing an adverse event unless the symptom worsened from baseline, or resolved and then recurred. For all adverse events of moderate or greater severity, additional information was captured, including the suspected relationship of the event to the study treatment. In the ten studies evaluating treatment of single episodes of malaria, relationship was classified as not related, or unlikely, possibly, probably, or definitely related to study medications. In the Kampala longitudinal study, the classification was similar, but the 'unlikely' category was excluded. Data were double-entered individually for each study into EpiInfo version 6.04 or Access (Microsoft Corporation, Redmond, Washington) and analysed using Stata version 8.0 (Stata Corporation, College Station, TX, USA). Categorical variables were compared using the chi-square test or Fisher's exact test. Continuous variables were compared using two-sample t-tests.

### Study population and summary of adverse events

A total of 5,614 study treatments were evaluated in 4,876 patients enrolled in the eleven clinical trials (Table 1). Serious adverse events were uncommon, and no important neurological events or severe dermatologic reactions were detected. Convulsions were the most commonly reported serious adverse event, followed by vomiting (Table 1). Of the 103 serious adverse events reported, none were classified as definitely related to treatment; most were considered to be unrelated (14 events), unlikely related (36 events), or possibly related (48 events) to the study medications (data not shown). Of the five serious events that were classified as probably related, all were episodes of vomiting. Two deaths occurred in the studies. One patient died of suspected severe malnutrition, and one patient died of congestive heart failure due to a presumed congenital heart defect (both patients had received AQ+SP).

Two important laboratory abnormalities were detected in the Kampala studies (Table 2). In the first Kampala study, one patient successfully treated with AQ+SP developed life-threatening thrombocytopaenia, neutropaenia, and anaemia, which were considered possibly related to the study medications [16]. Severe thrombocytopaenia (33,000/ul) was present at enrolment, persisted at day 7 (32,000/ul), and worsened by day 28 (20,000/ul). Severe neutropaenia and anaemia developed, and the child became transfusion-dependent. A bone marrow biopsy revealed hypoplastic marrow of undetermined cause with all cell lines present. The child survived, but the aetiology of the pancytopaenia was not identified. In the longitudinal Kampala study, one child developed repeated episodes of neutropaenia (four mild, one moderate, one severe) after AS+AQ treatment and was subsequently withdrawn from the study.

**Table 2:** Summary of studies comparing CQ+SP, AQ+SP, and AS+AQ demonstrating variability between study sites

**Trial**	**Kampala 1**	**UMSP 2**			
Study site	Kampala	Jinja	Arua	Tororo	Apac
Total participants enrolled	400	543	534	541	542
Median age years (IQR)	4.2 (+/- 4.6)	3.6 (+/- 5.5)	1.5 (+/- 1.4)	1.3 (+/- 1.5)	1.8 (+/- 1.9)
Age < 5 years	221 (55%)	332 (61%)	497 (93%)	500 (92%)	489 (90%)
Mean Hb Day 0 (SD)	10.2 (1.9)	10.6 (2.3)	9.3 (1.7)	9.0 (1.9)	9.3 (1.9)
Medications taken in past 2 weeks	167 (41%)	322 (59%)	83 (16%)	212 (39%)	196 (36%)
≥ 3 Additional medications prescribed	N/A	100/543 (18%)	150/534 (28%)	385/541 (71%)	50/542 (9%)

Risk of clinical treatment failure					
CQ+SP	59/126 (47%)	128/160 (46%)	50/178 (72%)	116/162 (72%)	52/180 (29%)
AQ+SP	18/130 (14%)	24/173 (14%)	73/174 (42%)	73/172 (42%)	28/178 (16%)
AQ+AS	16/130 (12%)	24/181 (13%)	62/171 (36%)	93/181 (51%)	46/172 (27%)
Mean AEs (SD) per participant	4.6 (3.3)	2.5 (2.2)	4.3 (2.2)	5.8 (2.5)	3.6 (2.2)

Participants reporting AEs (%)	371/396 (94)	438/540 (81)	521/534 (98)	534/541 (99)	522/541 (96)
CQ+SP	127/131 (97)	149/168 (89)	174/180 (97)	166/166 (100)	181/185 (98)
AQ+SP	120/132 (91)	143/185 (77)	176/180 (98)	178/181 (98)	175/182 (96)
AQ+AS	124/133 (93)	146/187 (78)	171/174 (98)	190/194 (98)	166/174 (95)

Total AEs by severity (%)	N = 1828	N = 1348	N = 2287	N = 3117	N = 1955
Mild	878 (48)	756 (56)	1736 (76)	1462 (47)	1322 (68)
Moderate	898 (49)	557 (41)	529 (23)	1622 (52)	613 (31)
Severe	48 (3)	30 (2)	21 (1)	31 (1)	20 (1)
Life-threatening	4 (0.2)	5 (0.4)	1 (0.4)	2 (0.1)	0

Total AEs by relationship* (%)	N = 1828	N = 554	N = 551	N = 1655	N = 631
Unlikely	1129 (62)	292 (53)	424 (77)	1159 (70)	384 (61)
Possible	480 (26)	248 (45)	127 (23)	464 (28)	238 (38)
Probable	199 (11)	14 (3)	0	32 (2)	9 (1)
Definite	0	0	0	0	0

* At UMSP sites (Jinja, Arua, Tororo, Apac), data on relationship was collected only for adverse events of moderate or greater severity

### Variability between study sites

The baseline characteristics of the study populations differed between the study sites. To demonstrate this variability, data are presented for the five trials comparing CQ+SP, AQ+SP, and AS+AQ, which were conducted in Kampala, Jinja, Arua, Tororo, and Apac (Table 2). The other studies are not included as they did not compare the same regimens, and/or used slightly different study protocols. Significant differences in participant age, mean haemoglobin, prior treatment, administration of concomitant medications, and risk of clinical treatment failure were observed (p < 0.001 for all comparisons). In addition, differences in the pattern of adverse event reporting were also noted. The mean number of adverse events per participant ranged from 2.5 in Jinja to 5.8 in Tororo (p < 0.001), and most events were mild or moderate in severity. However, the severity grading appeared to vary between the sites with the proportion of events graded as mild ranging from 47% in Tororo to 76% in Arua (p < 0.001). The majority of events were classified as unlikely or possibly related to the study treatment at all sites, but classification of relationship also varied.

### Clinical treatment failure and analysis of adverse events

In the studies evaluating single episodes of malaria, all symptoms of recurrent malaria, whether due to recrudescence or new infection, were captured as adverse events. In the analysis, clinical treatment failure, due to recrudescence and new infections, was a confounder for some, but not all, adverse events. Affected events included fever, weakness, anorexia, vomiting and elevated temperature, all common symptoms of malaria. Clinical treatment failure did not appear to confound associations between study treatment and events which were less likely to be symptoms of malaria, including abdominal pain, diarrhoea, cough, pruritus, and coryza.

**Figure 2:**
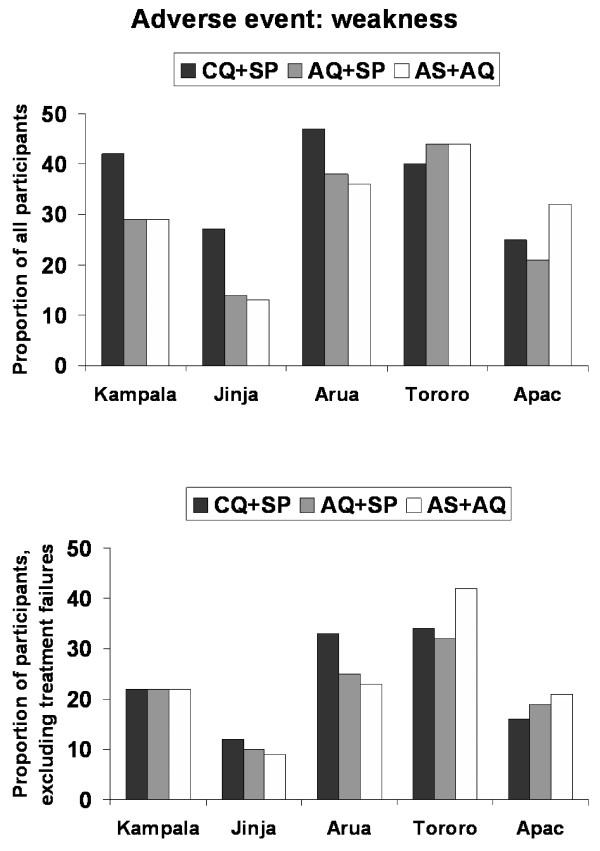
**Clinical treatment failure and analysis of adverse events**. The proportion of participants experiencing an adverse event of weakness in the five studies comparing chloroquine + sulphadoxine-pyrimethamine (CQ+SP), amodiaquine + sulphadoxine-pyrimethamine (AQ+SP), and artesunate + amodiaquine (AS+AQ) is shown for all participants, and after excluding clinical treatment failures.

Figure 2 illustrates this issue using the data collected for weakness in the five studies comparing CQ+SP, AQ+SP, and AS+AQ. Comparing the proportion of all participants in the treatment groups who developed an adverse event of weakness across the sites, significant differences were found in Kampala (p = 0.032), and in Jinja (p = 0.001), with CQ+SP associated with the highest risk of weakness. However, when the analysis was restricted by excluding clinical treatment failures, these differences were not sustained. In Kampala, the proportion of participants experiencing weakness fell from 42% to 22% in the CQ+SP group, and no difference was detected between the three treatment groups (p = 0.998). Similar results were seen in Jinja. Comparisons of risk of weakness between the treatment groups in the other sites were not significant for either analysis population. However, when clinical treatment failures were excluded the risk of weakness decreased in all groups at all sites.

### Challenges with data analysis

Analysis of the safety data presented several challenges. The adverse event data collected at the different sites was to be pooled in order to increase the power for detecting differences between the study treatments. However, the significant differences in baseline characteristics, transmission intensity, local treatment practices, and pattern of adverse event reporting at the different sites, limited the ability to effectively combine the data. The confounding effect of clinical treatment failure on the association between study treatments and adverse events was another challenge, as was the large number of adverse event outcomes and adverse events reported. To address this, several analytical approaches were considered. The analysis was restricted by excluding treatment failures and by excluding mild events. However, excluding treatment failures limited the available data, particularly in areas of high transmission. Excluding mild events also limited the data and the ability to assess for less severe events which might have an impact on adherence. A multivariate logistic regression model was also attempted to control for treatment failure and other potential predictors of adverse events. However, the number of adverse event outcomes and multiple comparisons still presented a challenge to the presentation and interpretation of the data.

## Discussion and evaluation

### Identifying and defining adverse events

Systematic assessment for adverse events, applying the broad ICH GCP definitions, was conducted in eleven antimalarial clinical trials. The substantial number of adverse event reports collected made evaluating for clinically important events, determining causality, and detecting relevant differences between antimalarial treatments, challenging. Although defining an adverse event as 'any untoward medical occurrence' ensures maximum sensitivity in capturing events, such a broad definition may not be ideal for antimalarial clinical trials. Assessment of adverse events in malaria studies is complicated by the overlap between symptoms of malaria and common drug side effects. The frequent occurrence of non-malarial illnesses, including viral infections and respiratory illnesses is also problematic, particularly in children.

### Clinical monitoring

Active assessment for specific symptoms and signs was conducted at all scheduled and unscheduled follow-up visits, rather than just relying on patient reports. This likely increased the number of adverse events detected, but allowed differences in tolerability to be captured, which may have been missed without systematic screening. Given the potential for neurotoxicity associated with artemisinins [23], simple neurological examinations were systematically performed in the studies. However, no important neurologic abnormalities were detected, raising the question of whether neurologic examinations should be routinely conducted in antimalarial clinical trials. Although routine screening is likely to be low yield, targeted assessments of neurological function, including audiometric testing for hearing, in populations at higher risk of adverse events such as young children and patients receiving repeated treatments may be more useful. Guidelines to standardize methods for assessing symptoms and conducting physical examinations are needed. Even subtle differences in the approach to defining symptoms, measuring temperature, and conducting physical examinations may affect the ability to pool data collected at different sites.

### Laboratory monitoring

Laboratory testing is an important component of safety monitoring. Recommendations on laboratory testing, specifically addressing the methodology and timing of follow-up evaluations, and which tests to conduct, would also be useful. In the Kampala studies, follow-up laboratory tests were conducted at different intervals due to differences in study design and requests of the study sponsors, limiting the ability to combine the data. Ideally, laboratory testing could be guided by the known pharmacokinetic and pharmacodynamic properties of the drugs, and standardized intervals for testing could be established. The choice of tests for routine screening should be guided by the drugs being assessed and any specific safety concerns. Once initial problems are detected with routine testing, additional diagnostic testing is often required. Even if sites have the capacity to perform routine tests, the capacity for more sophisticated assessments is often lacking. Establishing centralized laboratories where samples could be sent for more advanced testing would help support laboratory testing and safety monitoring.

### Grading severity of adverse events

In theory, severity grading should be the most objective parameter of adverse event reporting. However, clinical assessment and classification of severity may vary between physicians and between sites, even when a grading scale is applied. In addition, the severity categories for many symptoms are driven by the decision to treat; events that do not require treatment are classified as mild, while events that require treatment, intervention, and/or monitoring are considered moderate. Treatment decisions may also vary by site, and are likely to be different in Africa than in developed countries. Classifying more serious events as severe or life-threatening may also be subjective, particularly for clinical variables, and merging these two categories may help simplify severity grading.

### Methods of data capture

In an attempt to focus on more clinically relevant events, and to minimize the extra work associated with adverse event reporting, in most studies physicians were required to collect additional information, including suspected relationship, only on events of moderate or greater severity. This system may have provided an incentive for the study physicians to downgrade the severity classification. Variation in the proportion of events graded as 'mild' at the various sites (Table 2) would seem to support this, as greater consistency in severity grading between sites would be expected. Revising the system to capture data on all events, regardless of severity, may improve reporting, but will also generate even more data on potentially less relevant events. Focusing assessment on patient-reported events (rather than systematically screening for specific symptoms and exam findings), or limiting evaluation to serious adverse events and laboratory abnormalities, may also limit the data burden, but may underestimate differences in tolerability.

### Duration of follow-up

Standardized guidelines on the duration of follow-up for safety monitoring are also needed. The duration of follow-up for monitoring of adverse events after antimalarial therapy is variable, often coinciding with the recommended length of follow-up for efficacy outcomes of 28 or 42 days [5,6,13,15,24,25]. As for drug efficacy, the duration of adverse event assessment should be determined by the elimination half-life of the drugs under evaluation; however, the timing of active assessments for drug safety may be different than those for efficacy. In the longitudinal study in Kampala, few differences in the comparative risk of adverse events at 14 and 42 days of follow-up were found. In that study, active surveillance for adverse events was conducted until day 14, followed by passive surveillance. Detailed assessments for safety and tolerability conducted within the first two weeks of drug treatment are likely to have the greatest yield. Depending on the regimen, continued assessment for evidence of serious adverse events and laboratory toxicity may extend beyond that time.

### Determining causal associations between adverse events and treatment

Determining if an adverse event is causally related to treatment is a critical step in drug safety monitoring. However, establishing causal associations is challenging, and classification of relationship can be subjective and insensitive. In these studies, despite using a standard protocol and similar approaches to training, assessment of relationship lacked consistency. The majority of events (95%) were classified as unlikely or possibly related to the study medications; classification of an event as 'probably' related to the study treatment occurred rarely. This likely reflects the difficulty in ruling out alternative etiologies for an event, or an understandable reluctance on the part of physicians to commit to more definite relationship classes. Assessment for causality requires careful consideration of various factors including the timing of the event, the timing of the treatment, response to antimalarial therapy, presence of concomitant illnesses, and administration of concomitant medications and herbal therapies. Algorithms based on a decision tree considering these factors could be developed to help standardize assessment of the relationship between adverse events and antimalarial treatment.

### Analytical challenges: Limited sample size, pooling data, multiple comparisons

Antimalarial clinical trials are typically designed and powered to test hypotheses about efficacy. As a result, the sample size of trials generally limits the ability to detect uncommon events. The safety data collected at the different sites was to be pooled in order to increase the power to detect differences between the study treatments. However, significant differences in baseline characteristics, and patterns of drug use, were observed between the study sites. In addition, despite using a standardized protocol and similar training, differences in adverse event assessment and reporting at the different sites were observed, which complicated the analysis. The potential for such variation in baseline characteristics and adverse event monitoring may significantly impact on the ability to effectively combine data collected in different studies and sites, even if standardized protocols are used. Analysis of safety data is also challenged by the impact of multiple comparisons on hypothesis testing. The likelihood that significant differences between treatments may be found just by chance increases with the number of comparisons made, which may need to be taken into account when designing studies and interpreting results.

### Clinical treatment failure is a confounder

In the studies of single episodes of malaria, clinical treatment failure appeared to confound the association between study treatment and certain adverse events, particularly in areas of lower transmission intensity. A study participant who fails initial therapy and develops early treatment failure or recurrent clinical malaria will present with the signs and symptoms of acute illness, which will be captured as adverse events. In areas of high transmission, where participants are at risk of developing new infections within the study follow-up period, this will be an issue even if the drugs are highly efficacious. Thus, the efficacy of an antimalarial regimen for treating the initial infection, and for preventing new infections, will be associated with the risk of adverse events. One approach, which was considered, is to restrict the analysis by excluding treatment failures. However, excluding treatment failures limits the available data, particularly in areas of high transmission. Controlling for treatment failure and other relevant factors in a multivariate analysis is another approach. Alternatively, this problem could be addressed in the study design. In the longitudinal study of repeated malaria treatment episodes, a new cycle of adverse event reporting began with each new episode of malaria, and symptoms noted on that day were not considered adverse events but rather baseline for the new episode. However, the cohort study design may not be optimal for all settings.

## Conclusion

Further studies are needed to establish the safety profiles of newer antimalarial regimens in Africa, particularly with widespread use. Despite prospectively evaluating for adverse events using standardized methods modelled on currently available international guidelines, limitations in the system were identified. New guidelines for monitoring for safety and tolerability in antimalarial trials are needed. Although the WHO has acknowledged the need for pharmacovigilance, and has taken the lead in supporting the development pharmacovigilance systems in African countries deploying ACTs, additional guidance on adverse events monitoring in antimalarial clinical trials would be welcomed [2,26]. Such guidelines could include recommendations on methods of data collection, including laboratory testing, defining and identifying adverse events, grading severity, classifying relationship, and analyzing and reporting the data. Severity grading criteria could be standardized and revised to be more relevant to local settings, and algorithms for classifying relationship could be developed. Standardized guidelines for gathering safety data in antimalarial trials should specifically address how to detect events of greatest importance (including severe and life-threatening events, those with a causal relationship to the treatment, those which impact on adherence, and events not previously reported) while limiting the amount of data collected.

## Authors' contributions

SGS, PJR, GD, MRK, FWM, and AT conceived and designed the studies, SGS, AM, AY, HB, KB, CMS, TDC, DNM, BN and GD participated in data collection, SGS, PJ, HB, KB, and GD participated in the data analysis. All authors participated in the writing of the manuscript, read and approved the final manuscript. None of the authors declare any potential conflicts of interest, including financial interests and relationships and affiliations relevant to the subject of the manuscript.

## Supplementary Material

Additional file 1 Guidelines for physical examination. Click here for file

Additional file 2 Severity grading guidelines. Click here for file
