# Supplementary material for: Monitoring antimalarial safety and tolerability in clinical trials: A case study from Uganda
Source: Malar J. 2008 Jun 11;7:107. doi: 10.1186/1475-2875-7-107 (PMC2464601; doi:10.1186/1475-2875-7-107)
Supplement: Additional file 1 — Guidelines for physical examination. [file 1475-2875-7-107-S1.doc]

###### Additional File A. Guidelines for Physical Examination

| **Dehydration** | Assess skin touch and turgor, mucous membranes, eyes, crying, fontanelle, pulse, urine output |
| --- | --- |
| **Facial edema** | Assess for swelling of eyes, face, mouth |
| **Jaundice** | Assess for yellowing of the sclera. Also evaluate the palpepral conjunctiva, lips, and skin. |
| **Chest** | Observe the rate, rhythm, depth, and effort of breathing. Check the patient’s colour for cyanosis.  The maximum acceptable respiratory rate by age: **< 2 months = 60, 2-12 months = 50, 1-5 years = 40, above 5 years = 30.**  Inspect the neck for the position of the trachea, for supraclavicular retractions, and for contraction of the sternomastoid or other accessory muscles during inspiration.  Auscultate the anterior and posterior chest for normal breath sounds and any adventitious sounds (crackles or rales, wheezes, and rhonchi). *Crackles are intermittent, non-musical, fine or coarse sounds that may be due to abnormalities of the lungs (pneumonia, fibrosis, early congestive heart failure) or airways (bronchitis or bronchiectasis). Wheezes are high-pitched and result from narrowed airways. Rhonchi are relatively low-pitched and suggest secretions in large airways.*  If abnormalities are identified, evaluate for transmitted voice sounds. In addition, palpate the chest to assess for tactile fremitus, and percuss the chest to assess for areas of dullness*. Normal, air-filled lungs emit predominantly vesicular breath sounds, transmit voice sounds poorly with “ee” = “ee”, and have no tactile fremitus. Airless lung, as in lobar pneumonia, emits bronchial breath sounds, transmits spoken words clearly with “ee” = “aay” (egophany), and has an increase in tactile fremitus.* |
| **Abdomen** | Inspect and ausculate the abdomen. Listen for bowel sounds in the abdomen before palpating it. Palpate the abdomen in all 4 quadrants lightly and then deeply. Assess the size of the liver and spleen. To assess for peritoneal inflammation, look for localised and rebound tenderness, and voluntary or involuntary rigidity. |
| **Skin** | Inspect the skin for colour, turgor, moisture, and lesions. If lesions are present, note their location and distribution (diffuse or localised), arrangement (linear, clustered, annular, dermatomal), type (macules, papules, vesicles) and colour. |
| **Hearing** | In children < 4 years, test hearing by shaking a rattle or crinkling paper from behind the head of the child on the right and left. Note child’s reaction to the noise – inspecting for a startle, or turning of the head toward the direction of the noise.  In children > 4 years of age, estimate hearing by testing one ear at a time. Ask the patient to occlude one ear with a finger or, better still, occlude it yourself. Gently rub your thumb and index finger together within 2 inches or 5 cm from the patient’s unoccluded ear and assess if the patient is able to hear the noise.  In children of all ages, perform a full otoscopic exam if these screening methods reveal any diminunition of hearing. In patients > 4 years of age only, attempt to assess for conductive and sensorineural hearing loss, using the tuning fork. Set the fork into light vibration by tapping it. Place the base of the lightly vibrating tuning fork firmly on top of the patient’s head or midforehead and test for lateralization (Weber test). Ask where the patient hears it: on one or both sides. Normally, the sound is heard in the midline or equally in both ears. If nothing is heard, try again, pressing the fork more firmly on the head. In unilateral conductive hearing loss, sound is heard in (lateralized to) the impaired ear. Visible explanations include acute otitis media, perforation of the eardrum, and obstruction of the ear canal, as by cerumen. In unilateral sensorineural hearing loss, sound is heard in the good ear.  Next, to compare air conduction (AC) and bone conduction (BC) perform the Rinne test. Place the base of lightly vibrating tuning fork on the mastoid bone, behind the ear and level with the canal. When the patient can no longer hear the sound, quickly place the fork close to the ear canal and ascertain whether the sound can be heard again. Hear the “U” of the fork should face forward, thus maximising its sound for the patient*. Normally, the sound is heard longer through air than through bone (AC>BC). In conductive hearing loss, sound is heard through bone as long as or longer than it is through air (BC=AC or BC>AC). In sensorineural hearing loss, sound is heard longer through air (AC>BC).* |
| **Nystagmus** | Assess the extraocular movements in children of all ages by moving a torch or brightly coloured object in a circle, looking for conjugate movements of the eyes in each directions, or any deviation from normal and for nystagmus, a fine rhythmic oscillation of the eyes, analogous to a tremor in other parts of the body. *A few beats of nystagmus on extreme lateral gaze are within normal limits. If you see it, bring your finger in to within the field of binocular vision and look again*.  *The causes of nystagmus are multiple, including impairment of vision in early life, disorders of the labyrinth and the cerebellar system, and drug toxicity. Nystagmus occurs normally when a person watches a rapidly moving object. Nystagmus usually has both fast and slow movements, but is defined by its fast phase. For example, if the eyes jerk quickly to the patient’s left and drift back slowly to the right, the patient is said to have nystagmus to the left. The movements of nystagmus may occur in one or more planes (i.e. horizontal, vertical, or rotatory). It is the plane of the movements, not the direction of the gaze that defines this variable.* |
| **Tablet test** | For children > 9 months of age, ask the patient to pick a tablet (or equivalent object) up off a flat surface using the thumb and index finger of their dominant hand*. This tests for co-ordination of the upper extremity assessing the function of the motor system, cerebellar system, vestibular system (for coordinating eye and body movements) and the sensory system, for position sense. When testing small children, be aware that they will likely attempt to put the object into their mouth.* |
| **Heel-toe** | For patients > 4 years of age, asks the patient to walk heel-to-toe in a straight line – a pattern called tandem walking. If the patient is unable to perform this, ask them to walk across the room normally, and/or to walk on their toes and then on the heels, to further assess gait pattern. A gait that lacks coordination in instability is called ataxic and may be due to cerebellar disease or loss of position sense.  For children 2-4 years of age, assess gait by having them walk in a straight line. |
| **Romberg** | This should be performed in children > 4 years and is mainly a test of position sense. The patient should first stand with feet together and eyes open and then close both eyes for 20 to 30 seconds without support. Note the patient’s ability to maintain an upright posture. To test for pronator drift, have the patient stand for 20 to 30 seconds with both arms straightforward, palms up, and with eyes closed. A person who cannot stand may be tested for pronator drift in the sitting position. In either case, a normal person can hold this arm position well. Now, instructing the patient to keep the arms up and eyes shut, tap the arms briskly downward. The arms normally return smoothly to the horizontal position. This response requires muscular strength, coordination, and a good sense of position.  In ataxia due to loss of position sense, vision compensates for the sensory loss. The patient stands fairly well with eyes open, but loses balance when they are closed, a **positive Romberg sign**. In cerebellar ataxia, the patient has difficulty standing with feet together whether the eyes are open or closed. |

**1 February 2008**
