# Supplementary material for: Monitoring antimalarial safety and tolerability in clinical trials: A case study from Uganda
Source: Malar J. 2008 Jun 11;7:107. doi: 10.1186/1475-2875-7-107 (PMC2464601; doi:10.1186/1475-2875-7-107)
Supplement: Additional file 2 — Severity grading guidelines. [file 1475-2875-7-107-S2.doc]

**Additional File B. Severity grading guidelines**

**Table 1. Guidelines for Grading Patient Symptoms†**

|  | **Grade 1**  **MILD** | **Grade 2**  **MODERATE** | **Grade 3**  **SEVERE** | **Grade 4**  **LIFE THREATENING** |
| --- | --- | --- | --- | --- |
| **Subjective fever in the past 24 h** | N/A | Present (Yes) | N/A | N/A |
| **Weakness** | Mild decrease in activity, still playing | Moderate decrease in activity, playing limited | Not participating in usual activities, not playing | Lethargic |
| **Muscle and/or joint aches*** | Mild localised complaints | Mild diffuse complaints | Objective weakness; function limited | N/A |
| **Headache*** | Mild, no therapy required | Transient, moderate; therapy required | Severe; responds to initial narcotic therapy | Intractable; requires repeated narcotic therapy |
| **Anorexia** | Decreased appetite, but still taking solid food | Decreased appetite, avoiding solid food | Refusing to breast feed, appetite very decreased, no solids or liquids taken (< 2 years < 12 hr; > 2 years < 24 hr) | Refusing to breast feed, appetite very decreased, no solids or liquids taken (< 2 years < 12 hr; > 2 years < 24 hr) |
| **Nausea*** | Mild discomfort; maintains reasonable intake | Moderate discomfort; intake decreased significantly; some activity limited | Severe discomfort; no significant intake; activities limited | Minimal fluid intake |
| **Vomiting** | Transient emesis | Occasional or moderate vomiting | Orthostatic hypotension or IV fluids required | Hypotensive shock or hospitalization required for IV fluid therapy |
| **Abdominal pain*** | Mild | Moderate – no treatment needed | Moderate to severe – treatment needed | Severe – hospitalized for treatment |
| **Diarrhea** | Transient 3-4 loose stools/day | 5-7 loose stools/day | Orthostatic hypotension or > 7 loose stools/day or IV fluids required | Hypotensive shock or hospitalization for IV fluid therapy required |
| **Cough** | Transient – no treatment required | Continuous, requires treatment | Uncontrolled | Cyanosis, stridor, severe shortness of breath |
| **Pruritus** | Pruritus without rash | Pruritic rash, pruritus without rash that disturbs sleep | Mild urticaria | Severe urticaria, anaphylaxis, angioedema |
| **Tinnitus*** | Mild ringing or roaring sound | Moderate ringing or roaring sound | Severe ringing or roaring sound with associated hearing loss | N/A |
| **Behavioural changes** | Mild difficulty concentrating; mild confusion or agitation; activities of daily living unaffected; no treatment | Moderate confusion or agitation; some limitation of activities of daily living; minimal treatment | Severe confusion or agitation; Needs assistance for activities of daily living; therapy required | Toxic psychosis; hospitalization required |
| **“Flu”**  **(viral URI)** | Mild nasal congestion, mild rhinorrhea, no cough | Moderate nasal congestion, moderate rhinorrhea, cough present | N/A (if severe, classify individual symptoms) | N/A (if life-threatening, classify individual symptoms) |
| **Convulsion** | N/A | N/A | Localized or generalized seizure | Status epilepticus |
| *** Assess only in children > 3 years of age. Answer N/A for younger children and those unable to answer.** | | | | |

###### † Reference – Based on WHO Toxicity Grading Scale for Determining the Severity of Adverse Events

###### 1 February 2008

###### Table 2. Grading Physical Examination Findings

|  | **Grade 1**  **MILD** | **Grade 2**  **MODERATE** | **Grade 3**  **SEVERE** | **Grade 4**  **LIFE-THREATENING** |
| --- | --- | --- | --- | --- |
| **Temperature* (tympanic membrane)** | 38.0-38.4°C | 38.5-40.0°C | > 40.0°C | Sustained fever, equal or greater than 40.0°C for longer than 5 days |
| **Dehydration**  ****** | Normal skin turgor and touch, moist mucous membranes, tears present, eyes normal, fontanelle flat, CNS – consolable, pulse regular, urine output normal | Skin dry with + tenting, dry mucous membranes, eyes deep set, decreased tears, fontanelle soft, CNS – irritable, pulse slightly increased, urine output decreased | Skin clammy with lack of turgor, parched / cracked mucous membranes, sunken eyes, no tears, sunken fontanelle, CNS – lethargic, pusle increased, no urine output | |
| **Jaundice** | Slight yellowing of sclera and conjunctiva | Moderate yellowing of sclera and conjunctiva, yellowing of mucous membranes | Severe yellowing of sclera and conjunctiva, yellowing of skin | N/A |
| **Pallor** | Minimally pale conjunctiva, nail beds | Moderately pale conjunctiva, nail beds | Paper white conjunctiva, nail beds, palms. | N/A |
| **Eyes** | Redness, conjunctival injection, conjunctival discharge, excessive tearing | Eye pain, periorbital edema, exophthalmos, blurred vision, miosis, mydriasis, double-vision, | Blindness or visual field defecits, signs of endophthalnitis, paralysis of extraocular muscles, papilledema | N/A |
| **Ears** | Edema or hyperemia of pina, | Discharge from canal, tenderness or pina, red-swollen ear drum | Perforated ear drum | N/A |
| **Oropharynx** | Hyperemia, pigmentation, | Pharyngeal exudates or erythema | Tonsilar swelling, gum bleeding, blisters, ulceration | Tonsilar obstruction |
| **Facial edema** | Present, mild swelling of eyes | Moderate swelling of eyes, face | Severe swelling involving eyes, face, and mucous membranes; unable to open eyes | Airway compromise |
| **Neck** | Non-tender lymphadenopathy, erythema | Tender lymphadenopathy, swelling, tenderness, glandular enlargement | Tracheal deviation | Stridor |
| **Chest** | Mildly increased RR (for age, temperature), transient or localised adventitious sounds | Moderately increased RR, diffuse or persistent adventitious sounds | Rapid RR (< 2 months > 60, 2-12 months > 50, 1-5 years > 40, adults > 30)*nasal flaring, retractions | Cyanosis |
| **Cardio-vascular System (CVS)** | Grade 1 murmur | Asymptomatic change in rhythm or extra heart sounds (no treatment required); Grade 2 murmur | Recurrent/persistent change in rhythm or extra heart sounds (treatment required), Grade 3-4 murmur | Change in rhythm or extra heart sounds that require treatment and/or hospitalization; Grade 5-6 murmur |
| **Abdomen** | Normal bowel sounds, mild localised tenderness, and/or liver palpable 2-4 cm below the right costal margin (RCM), and/or spleen palpable, and/or umbilical hernia present | Normal or mildly abnormal bowel sounds, moderate or diffuse tenderness; and/or mild to moderately enlarged liver (4-6 cm below the RCM) and/or spleen palpable up to half-way between umbilicus and symphysis pubis | Severely abnormal bowel sounds, severe tenderness to palpation. Evidence of peritoneal irritation and/or significant enlargement of liver (> 6 cm below the RCM) and/or spleen palpable beyond half-way between umbilicus and symphysis pubis | Absent bowel sounds. Involuntary rigidity |
| **Skin†** | Localised rash, erythema, or pruritis | Diffuse, maculopapular rash, dry desquamation | Vesiculation, moist desquamation, or ulceration | Exfoliative dermatitis, mucous membrane involvement or erythema multiforme or suspected Stevens-Johnson or necrosis requiring surgery |

*** Reference - DMID Pediatric Toxicity Tables, May 2001, Drug Fever (Rectal)**

**** Reference – The Harriet Lane Handbook, 15^th^ edition, 2000**

**† Reference – WHO Toxicity Grading Scale for Determining the Severity of Adverse Events**

**1 February 2008**

###### Table 3. Grading Neurologic Examination Findings

|  | **Grade 1**  **MILD** | **Grade 2**  **MODERATE** | **Grade 3**  **SEVERE** | **Grade 4**  **LIFE-THREATENING** |
| --- | --- | --- | --- | --- |
| **Hearing** | *< 4 years: N/A*  > 4 years: Decreased hearing in one ear | *< 4 years: N/A*  > 4 years: Decreased hearing in both ears or severe impairment in one ear | *< 4 years: Any evidence of hearing impairment*  > 4 years: Severe impairment in both ears | N/A |
| **Nystagmus** | 3 or fewer beats of lateral nystagmus | More than 3 beats of lateral nystagmus | Sustained lateral nystagmus, any vertical or rotary nystagmus | N/A |
| **Tablet test** | Difficulty grasping tablet but able to pick up | Unable to pick up tablet without dropping | Unable to grasp tablet | N/A |
| **Heel-toe** | 2-4 years: Able to take at least 5 steps  > 4 years: Able to take at least 5 tandem steps | 2-4 years: Unable to take 5 steps  > 4 years: Unable to take 5 tandem steps | *2-4 years: Unable to walk*  > 4 years: Unable to perform tandem walk | N/A |
| **Romberg** | > 4 years: Unable to stand for 30 seconds with eyes closed | > 4 years: Unable to stand for 15 seconds with eyes closed | > 4 years: Unable to stand with feet together with eyes open or closed | N/A |
| **Clinical symptoms / sign** *(not otherwise specified)* | No therapy; monitor condition | May require minimal intervention and monitoring | Requires medical care and possible hospitalization | Requires active medical intervention, hospitalization, or hospice care |

**1 February 2008**

**Table 4.** Guidelines for Grading of Laboratory Abnormalities

|  | **Grade 1**  **MILD** | **Grade 2**  **MODERATE** | **Grade 3**  **SEVERE** | **Grade 4**  **LIFE-THREATENING** |
| --- | --- | --- | --- | --- |
| **Absolute neutrophil count* *(/****mm^3^****)*** | 750-1200 | 400-749 | 250-399 | < 250 |
| **Hemoglobin *(****g/dL)* | 9.0 – 9.9 | 7.0 – 8.9 | 5.0 – 6.9 | < 5.0 |
| **Platelets *(/****mm^3^****)**** | N/A | 50,000-75,000 | 25,000-49,999 | < 25,000 |
| **ALT *(U/L)***** | 1.1-4.9 x ULN  (50 – 224) | 5.0-9.9 x ULN  (225 – 449) | 10.0-15.0 x ULN  (450 – 675) | > 15.0 x ULN  (> 675) |
| **Bilirubin *(U/L)**** | 1.1-1.9 x ULN | 2.0-2.9 x ULN | 3.0-7.5 x ULN | > 7.5 x ULN |
| **Creatinine *(mg/dl))****  **Age < 2 years** | 0.6-0.8 | 0.9-1.1 | 1.2-1.5 | > 1.5 |
| **Creatinine *(mg/dl))****  **Age > 2 years** | 0.7-1.0 | 1.1-1.6 | 1.7-2.0 | > 2.0 |
| **Laboratory values** *(not otherwise specified)* | Abnormal but requiring no immediate intervention; follow | Sufficiently abnormal to require evaluation as to causality and perhaps mild therapeutic intervention | Sufficiently severe to require evaluation and treatment | Life-threatening severity; requires immediate evaluation, treatment, and usually hospitalization |

***Reference – DMID Pediatric Toxicity Tables, May 2001**

**** Reference – DAIDS Pediatric guidelines**

**1 February 2008**
